# Clinical acceptance and dosimetric impact of automatically delineated elective target and organs at risk for head and neck MR-Linac patients

**DOI:** 10.3389/fonc.2024.1358350

**Published:** 2024-03-14

**Authors:** Vesela Koteva, Björn Eiben, Alex Dunlop, Amit Gupta, Tarun Gangil, Kee Howe Wong, Sebastiaan Breedveld, Simeon Nill, Kevin Harrington, Uwe Oelfke

**Affiliations:** ^1^ Radiotherapy Physics Modelling, Division of Radiotherapy and Imaging, The Institute of Cancer Research, London, United Kingdom; ^2^ The Joint Department of Physics, The Royal Marsden Hospital and The Institute of Cancer Research, London, United Kingdom; ^3^ Head and Neck Unit, The Royal Marsden National Health Service (NHS) Foundation Trust and The Institute of Cancer Research, London, United Kingdom; ^4^ Head and Neck Unit, The Royal Marsden National Health Service (NHS) Foundation Trust, London, United Kingdom; ^5^ Department of Radiotherapy, Erasmus University Medical Center (MC) Rotterdam, Rotterdam, Netherlands; ^6^ Targeted Radiotherapy, Department of Radiotherapy and Imaging, The Institute of Cancer Research, London, United Kingdom

**Keywords:** clinical acceptability, dosimetric impact, MR-Linac, automated delineation, head and neck cancer

## Abstract

**Background:**

MR-Linac allows for daily online treatment adaptation to the observed geometry of tumor targets and organs at risk (OARs). Manual delineation for head and neck cancer (HNC) patients takes 45-75 minutes, making it unsuitable for online adaptive radiotherapy. This study aims to clinically and dosimetrically validate an in-house developed algorithm which automatically delineates the elective target volume and OARs for HNC patients in under a minute.

**Methods:**

Auto-contours were generated by an in-house model with 2D U-Net architecture trained and tested on 52 MRI scans via leave-one-out cross-validation. A randomized selection of 684 automated and manual contours (split half-and-half) was presented to an oncologist to perform a blind test and determine the clinical acceptability. The dosimetric impact was investigated for 13 patients evaluating the differences in dosage for all structures.

**Results:**

Automated contours were generated in 8 seconds per MRI scan. The blind test concluded that 114 (33%) of auto-contours required adjustments with 85 only minor and 15 (4.4%) of manual contours required adjustments with 12 only minor. Dosimetric analysis showed negligible dosimetric differences between clinically acceptable structures and structures requiring minor changes. The Dice Similarity coefficients for the auto-contours ranged from 0.66 ± 0.11 to 0.88 ± 0.06 across all structures.

**Conclusion:**

Majority of auto-contours were clinically acceptable and could be used without any adjustments. Majority of structures requiring minor adjustments did not lead to significant dosimetric differences, hence manual adjustments were needed only for structures requiring major changes, which takes no longer than 10 minutes per patient.

## 1 Introduction

**Figure 1:**
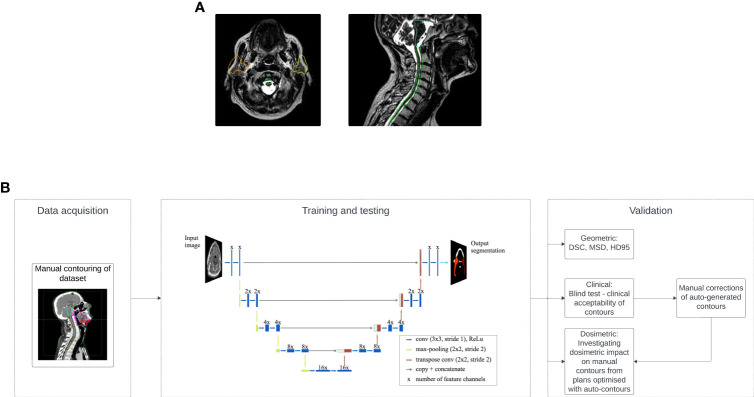
**(A)** Example of anatomical changes where contours of the parotid glands (orange: right parotid, yellow: left parotid), spinal cord (green) and brainstem (pale green) obtained from the initial scan are overlayed with the scan of the patient from the last fraction (fraction 30). **(B)** General workflow of the study split into three main groups: data acquisition, where a clinician manually contours all available data, training and testing where the model is trained to learn the manual contours and tested to produce a set of contours on an unseen scan, and last, validation where the model has been validated based on clinical acceptability, dosimetric impact and geometric analysis.

Every radiotherapy treatment starts with a generation of a treatment plan specifying a clinically optimized dose distribution and its delivery parameters for each patient. In our head and neck cancer (HNC) radiotherapy protocol, a treatment plan is initially generated based on a CT scan, prescribing 65 Gy to the primary target and 54 Gy to the elective target (the combined volume of the neck lymph nodes excluding the overlap of the nodes and the primary tumor) delivered in 30 fractions. The treatment plan is usually based on a CT scan, acquired one or more weeks before the treatment (1). However, this plan does not consider anatomical changes during treatment, risking compromised clinical goals and increased toxicity (1–7). For instance, the parotid glands may move closer to high-dose regions, posing a risk of overdosing these sensitive organs. **Figure 1A** shows a patient’s scan acquired during their final fraction (fraction 30) with overlayed contours of the parotid glands, spinal cord and brainstem from the patient’s initial scan. Hence, if we simply copy the contours from the initial to the final scan the parotid glands would be partially located outside of the patient’s external outline and the spinal cord and brainstem would not be at the correct position. Patient anatomy changes, even with radiotherapy masks, necessitate adapting contours to ensure accurate dose delivery.

Adaptive radiotherapy (ART) using an MR-Linac allows for real-time treatment plan adaptation based on daily anatomical changes (8, 9). In order to adapt the treatment plan, the ROIs need to be re-delineated on the daily scan while the patient is on the treatment couch. This requires organ delineation in less than one minute (10). Manual delineation, taking around 45 minutes, is infeasible for HNC patients within acceptable time frames. Current practice involves deformable image registration, but it requires initial manual delineation, lasting 45 to 75 minutes, and is prone to inaccuracies, often requiring additional manual adjustments due to imperfect results (11, 12).

In the past decade numerous groups have investigated automatic delineation using deep convolutional neural networks (CNNs) and have shown their great potential (13–16). The assessment of automatically generated contours typically relies on metrics like Dice similarity coefficient (DSC) and geometric measures such as Hausdorff distance. This study seeks to employ an in-house model for automatic delineation of the elective target volume and OARs, reducing delineation time for HNC patients, and assess the clinical acceptability and dosimetric impact of the auto-contours.

## 2 Materials and methods

A graphical representation of the complete workflow is shown in **Figure 1B**.

### 2.1 In-house model for automated delineation

The dataset employed in this study comprised 52 MR-Linac scans, 14 T1-weighted and 38 T2-weighted, obtained from 52 patients diagnosed with HNC. All scans were acquired using the 7MV (flattening filter free - FFF) Elekta Unity MR-Linac (Elekta AB, Stockholm, Sweden) with magnetic strength of 1.5T. A radiation oncologist thoroughly examined all available scans for each of the 52 patients, selecting a single scan per patient based on optimal imaging quality. Each scan was resampled via SimpleITK (Insight Software Consortium) (17, 18) to cover the HN region with an in-plane resolution of 0.6 x 0.6 mm^2^, slice thickness of 1.1 mm and dimensions (x, y, z) = (768, 768, 420), where z represents the number of slices, while x and y represent the number of pixels of each slice. From the available scans, 38 originated from the MOMENTUM study (19) and 14 were provided by the Royal Marsden Hospital (RMH), UK. The radiation oncologist delineated the neck nodes (levels 1a-5), parotid glands, spinal cord, brainstem, inferior pharyngeal constrictor muscle (IPCM), superior and middle pharyngeal constrictor muscle (SMPCM) and mandible.

A deep convolutional neural network (CNN) was trained to reproduce the manually-delineated (ground truth) structures. The CNN had a typical 2D U-Net architecture with 58 layers in total including batch normalization and activation layers (**Supplementary Material** provides more details. The MR scans were fed into the network slice-by-slice (20). This approach makes use of deep learning using Python (version 3.7) and the open-source libraries Tensorflow (21) and Keras both Google, Menlo Park, California, United States (22). The model was trained utilizing the computational power of an NVIDIA Tesla V100 GPU. Although some studies favor 2.5D and 3D U-Nets over 2D U-Nets (23, 24), providing this extra information doesn’t consistently enhance accuracy (15). Additionally, 2D CNNs are more computationally efficient than 2.5D or 3D U-Nets, requiring fewer resources for processing. We believe the available MRI scans have sufficient resolution for the task. Training a 3D network would demand decreased resolution and spatial size, risking loss of important features. Furthermore, 2D U-Nets require less data and are less prone to overfitting than 3D U-Nets, potentially leading to better generalization.

Leave-one-out cross-validation was used (25). This technique takes all but one patients as input for training and uses the remaining patient for testing. This is repeated until predictions are made for all patients. All images were downsampled by a factor of 2 before being fed to the network which was trained for 40 epochs with a learning rate of 0.0001. We used the Dice loss, optimizing it with the Adam optimizer (26, 27). Data augmentation was applied through rotation within ±3°, zoom up to ±10% and vertical/horizontal shifts up to 10% of the original image size.

We timed the generation of contours on a 3D scan and evaluated geometrically using DSC, mean surface distance (MSD) and 95^th^ percentile Hausdorff distance (HD95). The DSC shows how good the overlap between the auto-generated and manual contour is (1 for complete, 0 for none). The MSD represents the mean distance between each point of the auto-contour to the closest point from the manual contour. HD95 measures the largest distance among the closest 95% of the points from both contours (28).

### 2.2 Clinical acceptance

A clinical acceptance test by a second oncologist with 13 years of clinical experience assessed 684 contours—half manual, half model-generated. About 57% of model contours had DSC above 0.8, while 9% scored below 0.6. For detailed breakdown of the exact number of structures from the different groups of DSC that were presented to the oncologist we refer the reader to the **Supplementary Data**. To perform a ‘blind test’ the oncologist had no prior knowledge which contours were manual and which were auto-generated. The same patient and contours were presented to the oncologist on two separate days without their knowledge. The oncologist stated if the contours are clinically acceptable and if not, they stated the level of adjustments required from 1 to 5 (1 = minimal adjustments, 5 = complete re-contouring), similar to the method presented in (29). Afterwards a detailed breakdown was performed to find how many model-generated contours from each DSC group were classed as clinically acceptable and requiring minor and major adjustments.

### 2.3 Dosimetric impact

The dosimetric impact of the model-generated structures was evaluated. Using our standard clinical template, new treatment plans were optimized using the automatically generated contours and compared to dose distributions derived from the ground truth contours for 13 of the patients. These 13 patients were chosen as contours of the primary target volume were available, whereas contours of the primary target were not provided for the remaining patient population. To create the elective planning target volume, a margin of 3 mm was used around the combined volume of the left and right neck nodes, subtracting the primary planning target volume. The dosimetric impact was evaluated on the neck nodes separately without adding a margin. As quality indicators we selected the mean dose delivered to the parotid glands, pharyngeal constrictor muscles (PCMs) and mandible, maximum dose delivered to spinal cord and brainstem, and dose delivered to 95% of the volume of the neck nodes for both automated and manual structures. The results are presented as the absolute differences between the two respective doses normalized as a percentage of the prescribed dose of 65 Gy. Our findings are separated into three groups:

1. dosimetric impact on structures classified as clinically acceptable
2. dosimetric impact on structures that required minor changes (levels 1-2)
3. dosimetric impact on structures that required major changes (levels 3+).

Furthermore, we asked the oncologist to perform the required adjustments manually for several patients and recorded the time it would take to amend the contours. We compared the dosimetric results to the average absolute dosimetric difference between dose delivered to manually delineated structures and the corresponding automated and later on manually adjusted contours.

**Figure 2:**
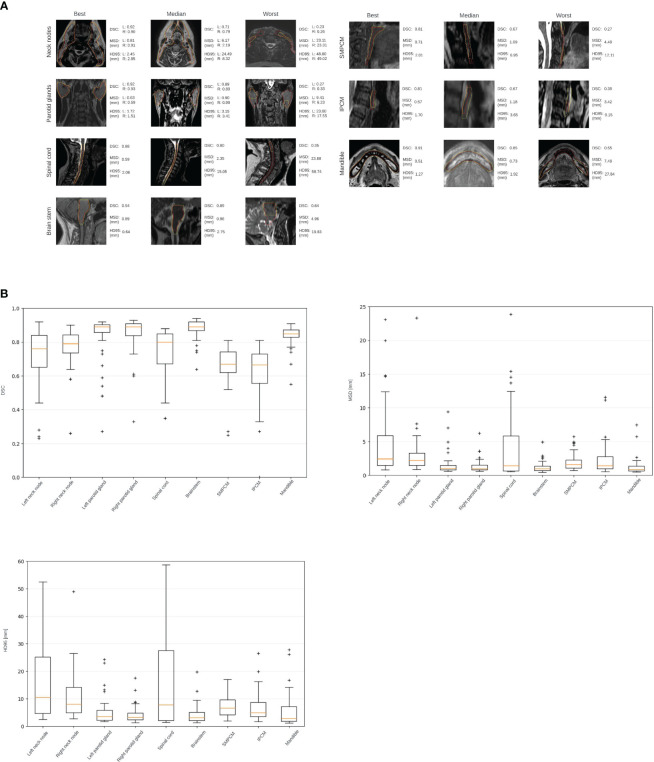
**(A)** Examples of the automatically generated contours (yellow) overlaid onto the manually delineated contours (red), representing the model's best, median, and worst performances determined by the DSC scores on a case-by-case basis. **(B)** Box plot showing the obtained range of the DSCs, MSDs, HD95s for all structures.

Approximate representation of the dosimetric impact for best, median, and worst algorithm performances was shown, analyzing cases from **Figure 2A**. The evaluation included median performance for neck nodes and parotid glands, worst and median performance for spinal cord, worst performance for brainstem, and worst, median, and best performance for mandible. Limited target volume information precluded analysis for other examples, so structures with similar DSC values were selected for assessment.

## 3 Results

### 3.1 In-house model for automated delineation

The network took an average of 32 hours to train (range 16-48 hours), while full 3D MRI organ delineation completed within 8 seconds. **Figure 2A** displays model-generated structures overlaying manually delineated contours, showcasing best, median, and worst performances based on DSC on a contour-by-contour basis. Average DSCs were 0.71 ± 0.17/0.77 ± 0.11, 0.84 ± 0.12/0.85 ± 0.10, 0.75 ± 0.13, 0.88 ± 0.06, 0.66 ± 0.11, 0.63 ± 0.15, and 0.84 ± 0.06 for left/right neck nodes, left/right parotid glands, spinal cord, brainstem, SMPCM, IPCM, and mandible, respectively. Further details on DSC, MSD, and HD95 are in **Figure 2B**.

### 3.2 Clinical acceptance

**Figure 3:**
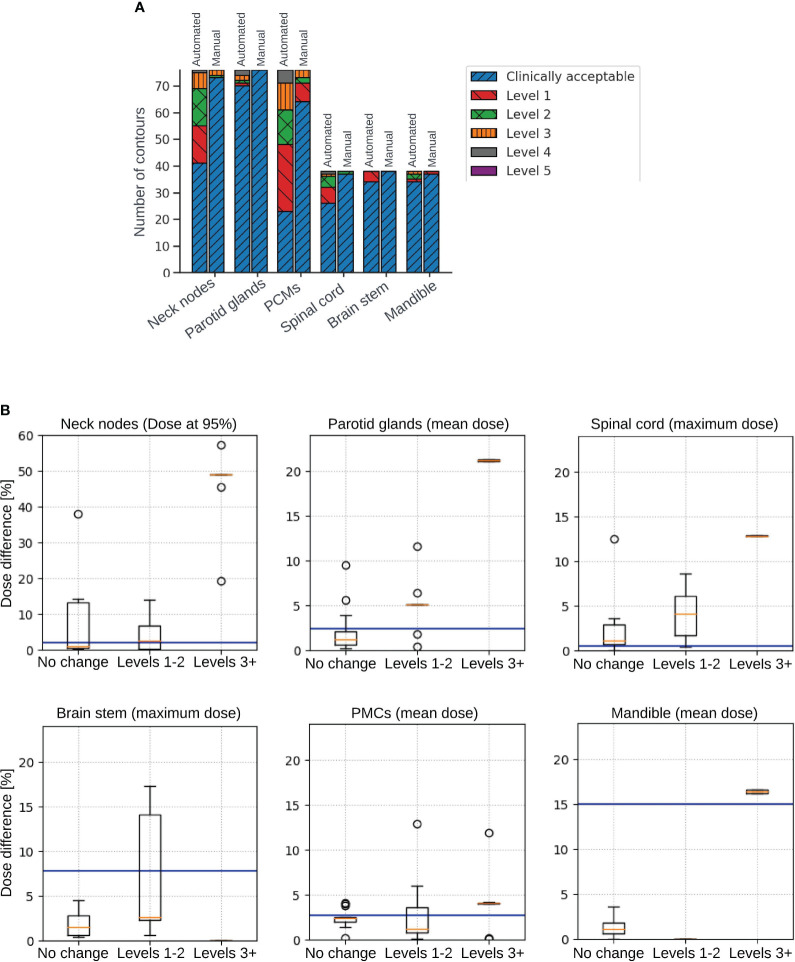
**(A)** Detailed breakdown of the number of automated and manual contours that were clinically acceptable, as well as requiring each level of corrections from 1 to 5. **(B)** Box plots illustrating dosimetric impact on structures that were clinically acceptable (not requiring any change), structures requiring minor changes (levels 1-2), and structures requiring changes of levels 3+. Plots show absolute difference in dose delivered to 95% of the volume of the neck nodes (top left), absolute difference in mean dose delivered to the parotid glands (top middle), absolute difference in maximum dose delivered to the spinal cord (top right), absolute difference in maximum dose delivered to the brainstem (bottom left), absolute difference in mean dose delivered to the pharyngeal constrictor muscles (PCMs) (bottom middle), and absolute difference in mean dose delivered to the mandible (bottom right). Blue horizontal lines represent the average dosimetric difference between dose delivered to algorithm-generated manually adjusted contours and manually delineated contours.

The blind test showed that 114 (≈ 33%) of the auto-generated contours required adjustments. The mean score of adjustments was $\bar{M}=\;1.89$ (range 1-4) with a median score of $\tilde{M}=\;1$ . From the manually delineated structures 15 (≈ 4.4%) required editing with mean score $\bar{M}=\;1.60$ (range 1-3) and median $\tilde{M}=\;1$ . **Figure 3A** shows detailed breakdown of the number of ROIs requiring adjustments.

**Figure 4:**
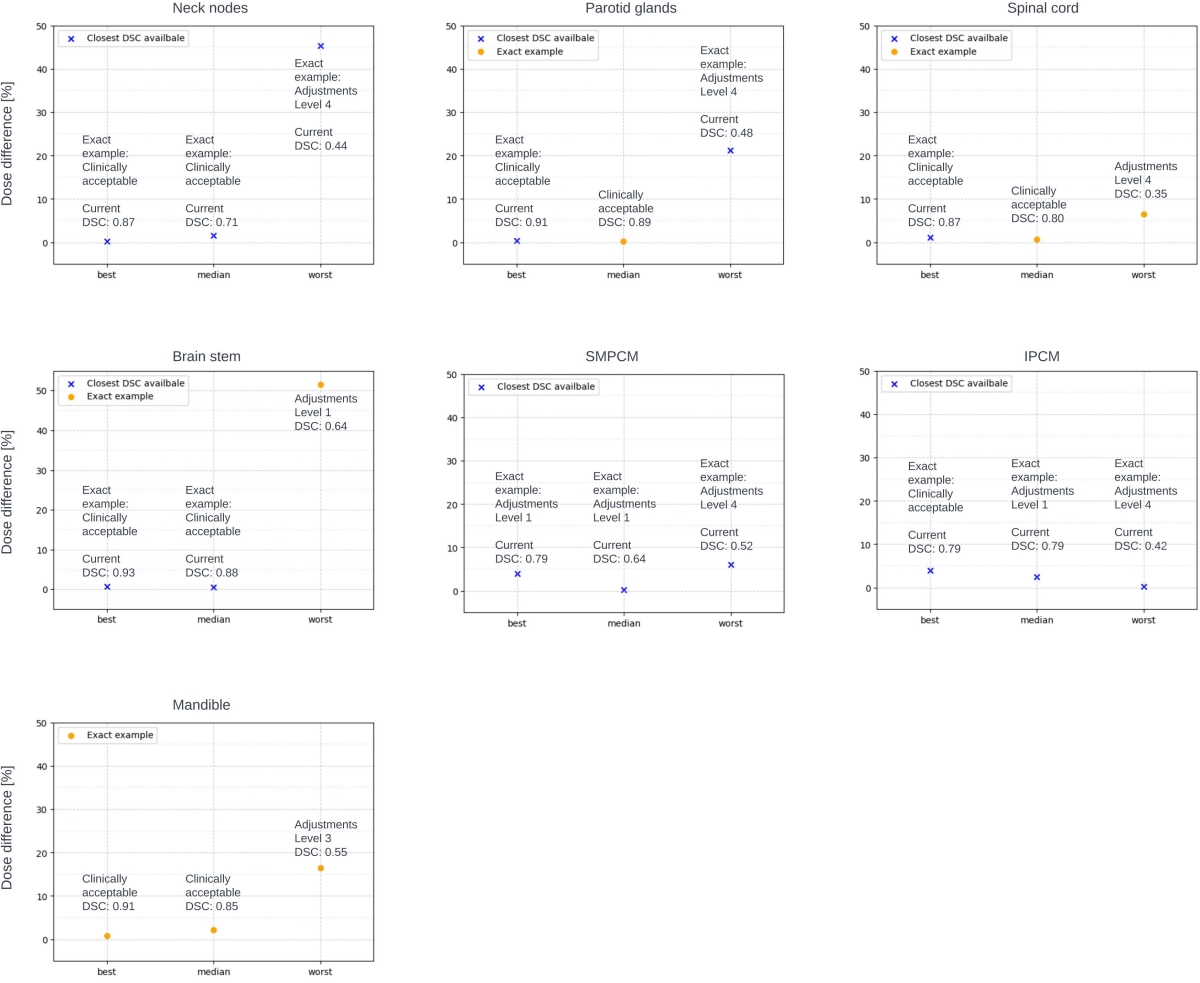
Clinical acceptance of exact examples from the best, median and worst performance of the model. Dosimetric difference evaluated for exact examples (orange circle) if information of the primary target was available, or with closest DSC (blue cross) to the exact example if primary target information was not available.

No clear DSC-adjustment correlation was observed. Generally, DSC*>*0.8 indicated clinically acceptable contours (except PCMs). DSC between 0.6 and 0.8 showed acceptability or minor changes (3 neck nodes needed major adjustments). DSC < 0.6 usually required major amendments. Due to the small size of the PCMs, most results scored DSC between 0.6 and 0.8 and majority were classed as requiring minor adjustments and when the DSC was below 0.6 majority of contours required major adjustments. Only 2 PCMs had DSC above 0.8 and one of them was clinically acceptable, whereas the other one required minor amendments. Of 196 contours with DSC≥0.8, 180 were acceptable, 16 needed minor adjustments; 39 out of 72 contours (DSC 0.7-0.8) were clinically acceptable, 32 needed minor adjustments, and one (neck node) required major changes. In the next group, 7 out of 43 structures (DSC 0.6-0.7) were acceptable, 31 needed minor adjustments, and 3 required major changes. Lastly, 2 out of 30 structures (DSC*<*0.6) were acceptable; 5 needed minor adjustments, and 23 required major changes. Detailed breakdown in **Supplementary Material**. Clinical acceptance test was performed for majority of the best, median and worst performance of the model except for the worst performance for neck nodes and best performance for the parotid glands. Based on the other results, most likely the neck nodes contours for the worst performance would have required major adjustments, while the best performance contours of the parotid glands would have been clinically acceptable. **Figure 4** shows detailed outcomes for other cases. All structures (except PCMs) from best and median performance were clinically acceptable; PCMs needed minor adjustments.

No correlation was found between amendment level and manual correction time. Average time for model-generated structure correction is 7.5 minutes per patient: 1min 27s for OAR with minor adjustments (levels 1-2) and 4mins 23s for neck nodes with the same adjustments level. For level 3+ adjustments, it takes 1min 4s for OAR and 4mins 39s for neck nodes. When presenting contours on two days, initially, the oncologist suggested level 1 corrections for SMPCM and right neck nodes, but later deemed all regions clinically acceptable.

### 3.3 Dosimetric impact

The results of the dosimetric impact analysis are shown in **Figure 3B**. The median absolute difference between dose delivered to auto-generated and manual contours for structures requiring no changes and structures requiring levels 1-2 amendments were very close and under 5% of prescribed dose. The average dosimetric difference between dose delivered to algorithm-generated, manually adjusted contours and manually delineated contours was in most cases higher than the median differences. Dosimetric difference was higher for structures requiring adjustments of levels 3+. **Figure 4** shows the dosimetric impact of the structure for which the model had best, median and worst performance or closest to these DSC values if information for the primary target was missing. DSCs of the structures used for this analysis were in the range of 0.44 - 0.87 for neck nodes, 0.48 - 0.91 for parotid glands, 0.35 - 0.87 for spinal cord, 0.64-0.93 for brainstem, 0.52 - 0.79 for SMPCM, 0.42 - 0.79 for IPCM, and 0.55 - 0.91 for Mandible. For all structures from the best and median performance of the model, the dosimetric difference is under 5% of prescribed dose.

## 4 Discussion

This study investigates the clinical acceptability and dosimetric impact of automatically obtained contours of the elective target volume and OARs required for treatment planning on MR-Linac HNC patients’ data. It was found that majority of automated contours (≈ 67%) were clinically acceptable and in general the ones that require minor adjustments do not lead to significant dosimetric differences.

With the increasing interest in deep learning-based strategies for automated segmentation in radiation oncology, numerous groups have developed their own in-house models. Kieselmann et al. (15) have developed a model, similar to the one presented in this study, for delineating the parotid glands on MRI, reporting average DSC of 0.85 ± 0.11, which is comparable to our achieved DSC of 0.84 ± 0.12*/*0.85 ± 0.10 for left/right parotid glands, respectively. Dai et al. (30) have also developed a very similar model for multi-organ delineation on MRIs, reporting again comparable results with average DSCs of 0.89 ± 0.06, 0.85 ± 0.06/0.86 ± 0.05, 0.77 ± 0.15 and 0.82 ± 0.10 for brainstem, left/right parotid gland, spinal cord and mandible, respectively. Their achieved average DSCs are marginally higher for the brainstem and the spinal cord, however they have not attempted to delineate the elective target volume. Korte et al. (31) and Kawahara et al. (32) have developed models to delineate the parotid gland and elective target volume levels II and III with Korte et al. investigating three different CNN, whereas Kawahara et al. compares CNNs to generative adversarial networks (GANs). Both groups achieve similar results for the parotid glands equivalent to the ones from the other discussed studies. Korte et al. have achieved 0.708 ± 0.053/0.715 ± 0.071 and 0.561 ± 0.100/0.573 ± 0.105 for left/right level II and level III, respectively. Kawahara et al. have shown that GANs have better performance when delineating the elective target volume with DSCs of 0.80/0.81 and 0.77/0.75 for left/right level II and level III, respectively. In comparison, we have achieved 0.71 ± 0.17/0.77 ± 0.11 for left/right neck node, however our contour is the combined volume of all levels 1a-5, therefore we cannot fairly compare our results.

Prior studies have predominantly focused on geometric evaluations, neglecting clinical acceptability. A 2020 review by Vrtovec et al. (33) highlighted this gap, emphasizing the scarcity of studies assessing the clinical viability of automated contours. Recent research post-2020 delves into the impact of auto-contours on HNC patient workflows (29, 34–36). Wong et al. (29) and Zhong et al. (35) mirrored a methodology similar to this study, seeking expert opinions on clinical acceptability or required adjustments for auto-contours. Zhong et al. found a majority of auto-contours clinically acceptable, aligning with this study, while Wong et al. reported that most required minor adjustments. Thor et al. (34) and Radici et al. (36) explored dosimetric impacts, with Thor et al. optimizing treatment plans using auto-generated contours and Radici et al. recalculating doses on auto-contours using original clinical plans. Notably, these studies utilized CT scans. Liu et al. (37) reviewed deep learning-based segmentation in the HN region, finding superior brainstem segmentation accuracy on MR scans (DSC 0.92) than CT (DSC 0.86). Other CT-based studies, He et al. (38) and Zhang et al. (39) reported successful auto-segmentation of HN region organs-at-risk (OARs). Our results showed improved parotid gland segmentation and comparable brainstem performance. Although spinal cord DSC was slightly lower, it remained comparable to inter-observer variability DSC. Strijbis et al. (40) segmented individual levels of the lymph nodes, achieving a combined structure DSC of 0.86, exceeding our model in geometrical evaluation. While the results showcase an impressive performance, it is noteworthy that the sizes of the available datasets for CT scans significantly surpass those for MR images. We expect an enhancement in the performance of our model as the dataset size expands. Moreover, these studies only reported geometrical results without dosimetric or clinical acceptability analyses. This study, to the best of current knowledge, is the first obtaining autocontours for elective target volumes and this set of crucial OARs (per clinical protocol) using MR-Linac HNC patient data. It specifically investigates both clinical acceptability and dosimetric impact, a facet rarely explored in prior research.

The geometric evaluation revealed lower DSC scores for neck nodes and PCMs. Larger HD95 values for the spinal cord and neck nodes suggested misclassified voxels and incomplete delineation. Instances with DSC below 0.6 led to increased Mean Surface Distance (MSD) and HD95 (see **Figure 2**). Detailed analysis highlighted misclassifications in various regions across multiple patients (left neck nodes for 10 patients, right neck nodes - 3 patients, left parotid - 4 patients, right parotid - 2 patients, SMPCM - 7 patients, IPCM - 16 patients, and mandible - 1 patient). Larger organs, like the neck nodes and spinal cord, resulted in higher maximum MSD and HD95 values due to their size. In some cases (refer to **Figure 2A**), correct classification was limited to specific areas, causing substantial differences in MSD and HD95 for lower DSC cases. The dataset’s mix of T1- and T2-weighted MRIs, with differing contrasts, most likely impacted the deep learning model’s performance. Studies have shown that when multiple oncologists delineate the neck nodes, the DSC ranges between 0.67 - 0.82 (41). Van der Veel et al. (42) have found that the mean DSC of the inter-observer variability is 0.82/0.83, 0.78, 0.88, 0.50/0.53/0.53, and 0.90 for left/right parotid glands, spinalcord, brainstem, superior/middle/inferior pharyngeal constrictor muscles, and mandible, respectively. Expectedly, due to their small size, PCMs had lower DSC values compared to other regions (43). However, the obtained value still closely aligns with the inter-observer variability DSC similar to the rest of the ROIs.

The blind test found that about 67% of model-generated and approximately 95.6% of manual contours were clinically acceptable. Approximately 75% of required adjustments for model-generated contours were only minor (mean level of changes: 1.89), with only around 8% of all aut-contours needing major adjustments (levels 3-4). The oncologist noted that PCMs are generally thin (3 mm), but slight widening was observed on number of presented examples. We explored the relationship between DSC metrics and clinical acceptability criteria, building on Heilemann et al.’s (44) suggestion of a DSC threshold above 0.7 for clinical acceptability. However, due to size-dependent characteristics, smaller ROIs may still be deemed acceptable with DSC below 0.7. Our results indicated that DSC below 0.6 tended to signify major adjustments, and while higher DSC values generally suggested better clinical acceptability, a straightforward correlation between DSC and adjustment levels was not apparent. Corrections didn’t consistently correlate with the time required for manual adjustments, averaging 7.5 minutes per patient for non-clinically acceptable contours. Evaluation time averaged about 1.5 minutes per patient for the oncologist. Therefore, the entire process — generating, evaluating, and potentially adjusting some structures - averages under 10 minutes per patient, significantly quicker than manual delineation. The different clinical acceptability outcome for the same patient on different days suggests subjectivity, potentially addressable with deep learning strategies.

The dosimetric impact revealed higher average absolute dosimetric differences for contours needing more adjustments, with outliers stemming from OAR proximity to high-dose regions and steep dose falloff. Achieving the clinical goal for the elective target volume heavily relies on precise contours; expanding the exposed range to cover any shape often meets goals but lacks clinical acceptability. Median dosimetric differences between clinically acceptable contours and those needing minor adjustments (levels 1-2) are quite similar. PCMs and neck nodes requiring minor adjustments show slightly lower median values than clinically acceptable ones. Except for the spinal cord, average dosimetric differences between algorithm-generated manually adjusted contours and manual delineated ones surpass median dosimetric differences between automated and manual contours. In most cases, contours needing minor changes can be used without significant dosimetric impact changes. Higher dosimetric differences are observed for contours needing level 3+ changes, representing only 8% of automated contours. Correcting these takes an average of about 1.5 minutes per organ, varying with organ size. Notably, the dosimetric analysis for best, median, and worst performance echoed general findings, highlighting an intriguing case where brainstem misclassification led to a significant dosimetric difference despite being categorized as needing only level 1 adjustments (see **Figure 2A**).

After thorough evaluation, we are confident in the algorithm’s effectiveness for contouring the parotid glands, brainstem, and mandible. While the outcomes for pharyngeal constrictor muscles were less satisfactory, a detailed dosimetric investigation showed minimal dosimetric differences in most cases. The algorithm shows promise for automating segmentation of the elective target volume and spinal cord, though additional refinements are needed for precision.

A key limitation in this and similar studies conducted on MR scans, is the limited availability of high-quality data. There are vast amounts of delineated CTs available, however consistent planning MRI data collection has only recently started. We utilized the entirety of the accessible data, resulting in a composite dataset with both T1 and T2-weighted MR-Linac scans. This combination may have negatively affected automated segmentation precision. Future research could explore using separate models for T1-weighted and T2-weighted scans, aiming for improved segmentation accuracy through such differentiation. Some of the other limitations of the current study were that primary target information was provided only for 13 of the patients. This allowed us to perform dosimetric analysis only for a small proportion of the patient population and cannot state for certain that the findings will remain the same when tested on larger patient population. Future studies would benefit of primary target information for all patients in order to perform more generalized dosimetric analysis. Furthermore, contouring of the primary target cannot be attempted with the current available data. Delineation of primary target varies among experienced clinicians and requires additional sequences (e.g., T1 post Gd or T2 SPAIR) along with endoscopic findings to aid contouring, accounting for natural anatomical barriers to tumor spread, such as air or bone. Another limitation is our reliance on contours delineated by a single oncologist as the ground truth. The clinical acceptability test showed that not all of these contours would be considered acceptable by another expert, highlighting the influence of inter-observer variability specifically, for smaller ROIs such as the pharyngeal constrictor muscles, the DSC is relatively low, ranging between 0.50 and 0.53. To enhance the model’s learning, incorporating contours from multiple experts would be beneficial. Furthermore, evaluating the results by only one oncologist could lead to personal bias. Therefore, incorporating clinical acceptability evaluations by multiple different experts for each task could offer a robust solution to enhance the validity and reliability of our findings. However, our oncologists have been through multiple quality assurance exercises aligned with established international benchmarks, such as the Gregoire et al. (45) atlas for nodal contouring. This ensures the reliability of the ‘clear’ pass or fail outcome derived from this assessment.

## 5 Conclusion

Majority (67%) of contours of the elective target volume and organs at risk for HNC patients automatically generated by an in-house developed model were found to be clinically acceptable and could be used for treatment planning without any manual adjustments. Among structures categorized as unfit for clinical use, the majority (≈75%) required only minor adjustments and the dosimetric impact showed that not performing the changes did not lead to significant dosimetric differences in most scenarios. Significant dosimetric differences could be observed for this group only if the ROIs or parts of ROIs were located exactly at the steep dose gradient. The model reliably contoured the parotid glands, brainstem, and mandible. The outcomes for the pharyngeal constrictor muscles were acceptable and the dosimetric impact analysis reveals minimal differences in most cases. While the algorithm shows promise for automating segmentation of the elective target volume and spinal cord, refinements could be performed for acquiring required precision in these areas. The analysis for the structures requiring major adjustments led to the conclusion that the time required for these adjustments to be made is minimal (on average 1min 4s per OAR and 4mins 39s per nCTV). Thus, delineation for HNC patients could be significantly sped up and the presented model could be used for initial delineation and subsequent re-delineation for each treatment fraction.

## Data availability statement

The dataset (MRI scans) used for this article was provided by the MOMENTUM study and the Royal Marsden Hospital for which the authors have directly applied. The dataset itself is not presented in the article. All presented analysis is original and there are no restrictions that apply. Requests to access these datasets should be directed to the Data Management Task Force, MOMENTUM@lygature.org.

## Ethics statement

Ethical approval was not required for the study involving humans in accordance with the local legislation and institutional requirements. Written informed consent to participate in this study was not required from the participants or the participants’ legal guardians/next of kin in accordance with the national legislation and the institutional requirements. MOMENTUM (NCT04075305) stands as an observational cohort study, operating as a collaborative effort between various institutions globally and Elekta (Stockholm, Sweden). Approval from the Institutional Review Board (IRB) was obtained at each center involved. Patients were provided with the opportunity to consent to the collection of health-related quality of life (HRQOL) data.

## Author contributions

VK: Conceptualization, Data curation, Formal analysis, Investigation, Methodology, Software, Writing – original draft. BE: Software, Supervision, Writing – review & editing. AD: Conceptualization, Supervision, Validation, Writing – review & editing. AG: Data curation, Writing – review & editing. TG: Writing – review & editing. KW: Validation, Writing – review & editing. SB: Writing – review & editing. SN: Supervision, Writing – review & editing. KH: Supervision, Writing – review & editing. UO: Funding acquisition, Supervision, Writing – review & editing.
