# Supplementary material for: Clinical acceptance and dosimetric impact of automatically delineated elective target and organs at risk for head and neck MR-Linac patients
Source: Front Oncol. 2024 Mar 14;14:1358350. doi: 10.3389/fonc.2024.1358350 (PMC10976943; doi:10.3389/fonc.2024.1358350)
Supplement: Supplementary file 1 [file DataSheet_1.pdf]

Supplementary Material

In-house developed CNN structure

The in-house developed deep learning CNN algorithm consists of a total of 58 layers with detailed breakdown as follows:

- **Downsampling Path (Encoder):** 3 levels * (2 conv + 2 batch norm + 2 ReLU) + 3 MaxPooling2D = 21 layers
- **Bottleneck:** 2 conv + 2 batch norm + 2 ReLU = 6 layers
- **Upsampling Path (Decoder):** 3 levels * (3 conv + 3 batch norm + 3 ReLU) + 3 UpSampling2D = 30 layers
- **Final Prediction Layer:** 1 Conv2D = 1 layer

Table S1. Detailed breakdown of the number of structures that achieved Dice similarity coefficient (DSC) higher than or equal to 0.8, between 0.7 and 0.8, between 0.6 and 0.7, and less than 0.6.

	DSC >= 0.8	0.8 >DSC >= 0.7	0.7 >DSC >= 0.6	DSC <0.6
Neck nodes	42	22	8	4
Parotid glands	65	6	1	4
Spinal cord	20	9	5	4
Brain stem	34	3	1	0
PCMs	2	30	27	17
Mandible	33	3	1	1

Table S2. Detailed breakdown of the number of structures from each DSC group that were classed as 'clinically acceptable', 'requiring minor adjustments of level 1-2', and 'requiring major adjustments of level 3+'.

	DSC >= 0.8			0.8 >DSC >= 0.7			0.7 >DSC >= 0.6			DSC <0.6		
	Clin. acc.	Level 1-2	Level 3+	Clin. acc.	Level 1-2	Level 3+	Clin. acc.	Level 1-2	Level 3+	Clin. acc.	Level 1-2	Level 3+
Neck nodes	33	9	0	8	13	1	0	66	12	0	0	4
Parotid glands	64	1	0	6	0	0	0	1	0	0	0	4
Spinal cord	18	2	0	8	1	0	0	5	0	0	2	2
Brain stem	32	2	0	2	1	0	0	1	0	0	0	0
PCMs	1	1	0	13	17	0	7	17	3	2	3	12
Mandible	32	1	0	2	1	0	0	1	0	0	0	1
